# Mitochondria’s Role in the Maintenance of Cancer Stem Cells in Glioblastoma

**DOI:** 10.3389/fonc.2021.582694

**Published:** 2021-02-22

**Authors:** Yasaman Iranmanesh, Biao Jiang, Okoye C. Favour, Zhangqi Dou, Jiawei Wu, Jinfan Li, Chongran Sun

**Affiliations:** ^1^ School of Medicine, Zhejiang University, Hangzhou, China; ^2^ Department of Radiology, The 2nd Affiliated Hospital of Zhejiang University Medical School, Hangzhou, China; ^3^ Department of Neurosurgery, The 2nd Affiliated Hospital of Zhejiang University Medical School, Hangzhou, China; ^4^ Department of Pathology, The 2nd Affiliated Hospital of Zhejiang University Medical School, Hangzhou, China

**Keywords:** glioblastoma, GSC, stem cell, mitochondria, quiescence, metabolism, stemness

## Abstract

Glioblastoma (GBM), one of the deadliest primary brain malignancies, is characterized by a high recurrence rate due to its limited response to existing therapeutic strategies such as chemotherapy, radiation therapy, and surgery. Several mechanisms and pathways have been identified to be responsible for GBM therapeutic resistance. Glioblastoma stem cells (GSCs) are known culprits of GBM resistance to therapy. GSCs are characterized by their unique self-renewal, differentiating capacity, and proliferative potential. They form a heterogeneous population of cancer stem cells within the tumor and are further divided into different subpopulations. Their distinct molecular, genetic, dynamic, and metabolic features distinguish them from neural stem cells (NSCs) and differentiated GBM cells. Novel therapeutic strategies targeting GSCs could effectively reduce the tumor-initiating potential, hence, a thorough understanding of mechanisms involved in maintaining GSCs’ stemness cannot be overemphasized. The mitochondrion, a regulator of cellular physiological processes such as autophagy, cellular respiration, reactive oxygen species (ROS) generation, apoptosis, DNA repair, and cell cycle control, has been implicated in various malignancies (for instance, breast, lung, and prostate cancer). Besides, the role of mitochondria in GBM has been extensively studied. For example, when stressors, such as irradiation and hypoxia are present, GSCs utilize specific cytoprotective mechanisms like the activation of mitochondrial stress pathways to survive the harsh environment. Proliferating GBM cells exhibit increased cytoplasmic glycolysis in comparison to terminally differentiated GBM cells and quiescent GSCs that rely more on oxidative phosphorylation (OXPHOS). Furthermore, the Warburg effect, which is characterized by increased tumor cell glycolysis and decreased mitochondrial metabolism in the presence of oxygen, has been observed in GBM. Herein, we highlight the importance of mitochondria in the maintenance of GSCs.

## Introduction

Glioblastoma (GBM) is the most common primary brain malignancy and is characterized by a variable survival time ranging from 4 to 16 months, depending on the status and the type of therapy the patients receive. Unlike most other types of malignancies, distant or extraneural metastasis of GBM is rare (1). However, GBM remains one of the incurable primary brain malignancies due to several factors. For instance, the absence of a single targetable oncogenic pathway is one of the contributing factors that further complicate the course of GBM treatment and research. GBM resistance to temozolomide (TMZ), a principal first-line chemotherapeutic agent, is mediated through several pathways and mechanisms. These include, methylguanine-DNA-methyltransferase (MGMT) (2, 3), long non-coding RNAs such as lncRNA TP73-AS1 (4), increased angiogenesis (5), resistance to apoptosis and apoptosis-inducing agents (6), mitochondrial DNA mutation, and most importantly, the presence of GBM initiating cells (GICs). According to Gimple et al., GICs are a heterogeneous population of GBM cells formed by the mutation of neural progenitor cells, immature neural stem cells (NSCs), or mature cells such as neurons. GICs give rise to glioblastoma stem-like tumor-initiating cells (GSLTICs) and their smaller subpopulation, GSCs, which are known to be the leading cause of GBM therapy resistance (7, 8). Interestingly, not only GSCs but also other subpopulations of GBM cells (such as GSLTICs) are capable of displaying stem cell properties (7). In response to microenvironmental changes such as hypoxia, these cells undergo a “state” transition and display phenotypic adaptation resulting from intrinsic tumor plasticity. In summary, plasticity imposed by microenvironment will determine the fate of the original GSC. Plasticity may also be responsible for reprogramming committed GBM progenitor cells and differentiated GBM cells to dedifferentiate into GSCs (8). It is noteworthy that the terms glioblastoma stem-like cells (GSLCs) and GSCs are vaguely described and used interchangeably in various reports. However, in our report, we introduce a three compartment model comprising; a) GSCs that are quiescent, self-renew slowly or infrequently and have the potential to proliferate, whereas GSLCs are proliferating GSCs that can self-renew under certain conditions, b) glioblastoma progenitor cells that proliferate rapidly and are committed to differentiate, and c) differentiated GBM cells. GSLCs are similar to progenitor cells in that, they are dedicated to differentiate and proliferate. Regarding metabolism, GSCs exhibit flexibility compared to neural stem cells due to the presence of certain enzymes [like pyruvate kinase isozyme 1 (PKM1) and pyruvate kinase isozyme 2 (PKM 2)] that enable GSCs to switch between glycolysis and oxidative phosphorylation (9). Both mitochondrial function and dysfunction play a significant role in GBM tumorigenesis, as mitochondria modulate the maintenance of GBM stemness, quiescence, and differentiation, whereas mitochondrial impairment is essential in arbitrating GSCs’ resistance to treatment. Previous studies during the last decades have not been successful in resolving this issue. That said, understanding the involvement of mitochondria in GSC quiescence might shed some light on GBM pathophysiology. This review emphasizes the importance of mitochondria in maintaining GSC stemness, quiescence, and metabolism. Also, we highlight the general features of GSCs, GBM progenitor, and differentiated GBM cells.

### Research History on Cancer Stem Cells

The history of cancer stem cell (CSC) research goes back to 1994 when leukemia initiating cells were identified (10). Identification of CSCs was a major breakthrough that could explain highly recurrent malignancies, such as GBM. Primarily, the extent to which oncogenesis and metastasis involve CSCs is unknown; however, as we learned more about CSCs in different types of malignancies such as liver, colorectal, ovarian, and brain cancers (for example, GBM), we realized how important these cells could be for an effective targeted cancer therapy. CSCs, characterized by their unique self-renewal and differentiating capacity, generate various tumor cells with different genetic constitutions, such as new GSCs and GBM neural progenitor cells that, in turn, give rise to the differentiated cells. The ability to stay in the quiescent state (during the G0 phase of the cell cycle) allows them to survive during the intensive cancer treatment. Recent discoveries have attributed glioblastoma resistance to the presence of cancer stem cells or so-called glioblastoma stem cells (GSC). GSCs, which originate from malignant transformation of neural stem cells (NSCs) of the subventricular zone (SVZ) tissues and differentiated neural cells such as astrocytes, maintain GBM tumor heterogeneity (11, 12).

### General Features of GSCs

**Figure 1:**
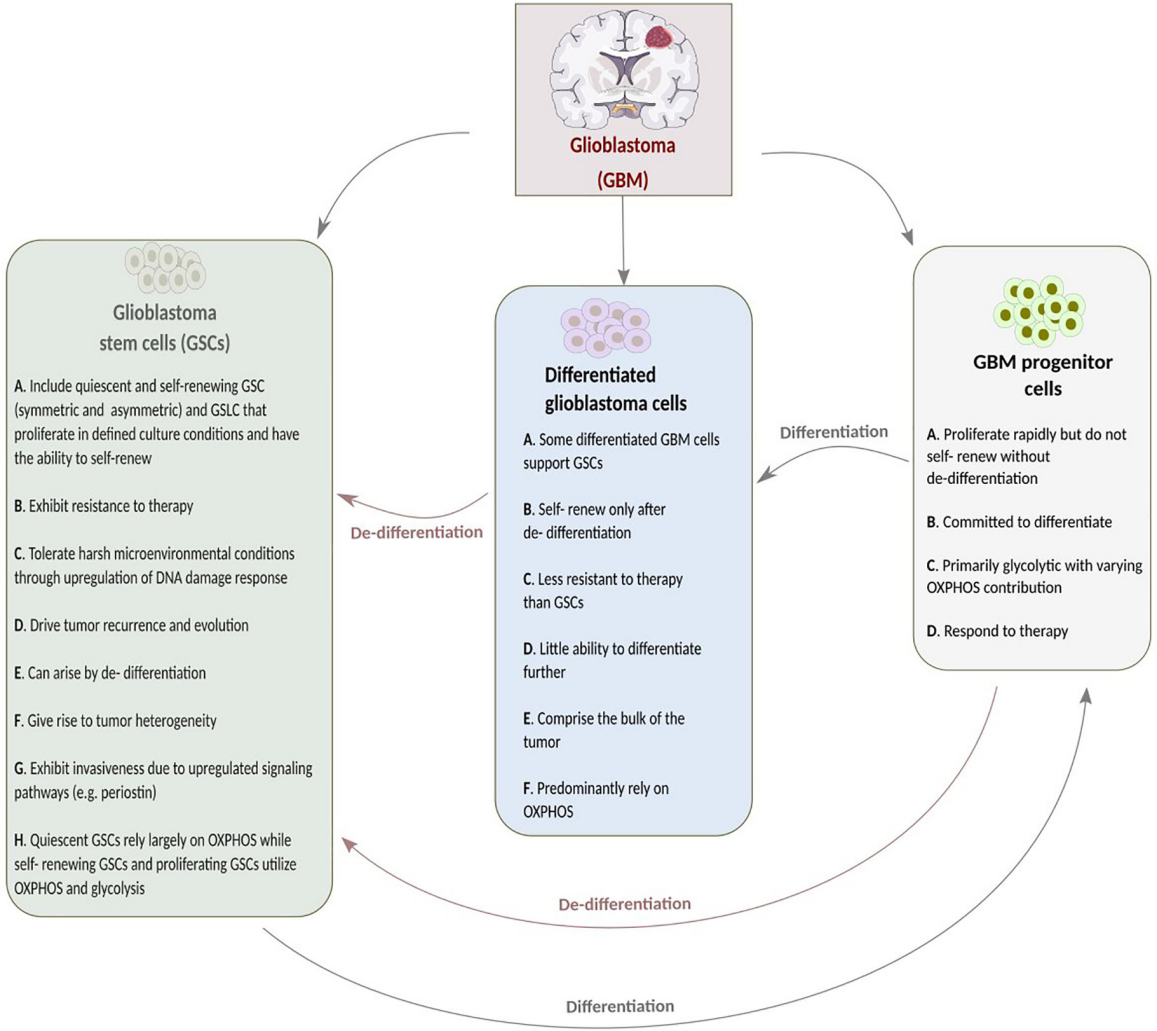
A summary of the features of glioblastoma stem cells (GSCs), glioblastoma (GBM) progenitor cells and differentiated glioblastoma stem cells.

GSCs are distinguished from neural stem cells by their molecular, genetic, metabolic, and dynamic features. Cancer stem-like cells have fragmented mitochondria compared to differentiated GBM cells, which possess tubular-shaped mitochondria (13, 14). Stem cells are said to have fewer and less mature mitochondria that are relatively inactive compared to those of differentiated cells, resulting in decreased ROS generation and, thus, low ROS levels required for the maintenance of stem cell quiescence and self-renewal potential (15, 16). Previously, it was said that CSCs might favor glycolysis as it regulates stemness and minimizes ROS generation (17). However, recent studies suggest quiescent CSCs depend largely on OXPHOS. This is also true for differentiated non-proliferating GBM cells that cannot further differentiate. On the other hand, proliferating CSCs utilize both glycolytic and oxidative pathways. Depending on oxygenation, nutrient availability and tumor microenvironment, proliferating GSCs can transition between glycolytic and oxidative pathways (7, 18, 19). CSCs utilize both glycolysis and OXPHOS since they switch between quiescent and proliferation “states.” A study on human TS1 GSLCs, upon acidic pH shift-induced quiescence, demonstrated the remodeling of mitochondria from tubular to donut shape to corroborate this. Similarly, placing the quiescent cells in a less acidic environment induced the alteration of mitochondria from donut to tubular shape (20). This study not only implies that donut-shaped mitochondria might be a feature of quiescent GSLCs but also suggests that mitochondria shape and function is dependent on GSLCs microenvironment. The influence of tumor microenvironment on CSCs has been extensively discussed elsewhere (14). Other features of GSCs and differentiated glioblastoma cells are shown in **Figure 1**.

#### Molecular and Genetic Features of GSCs

**Figure 2:**
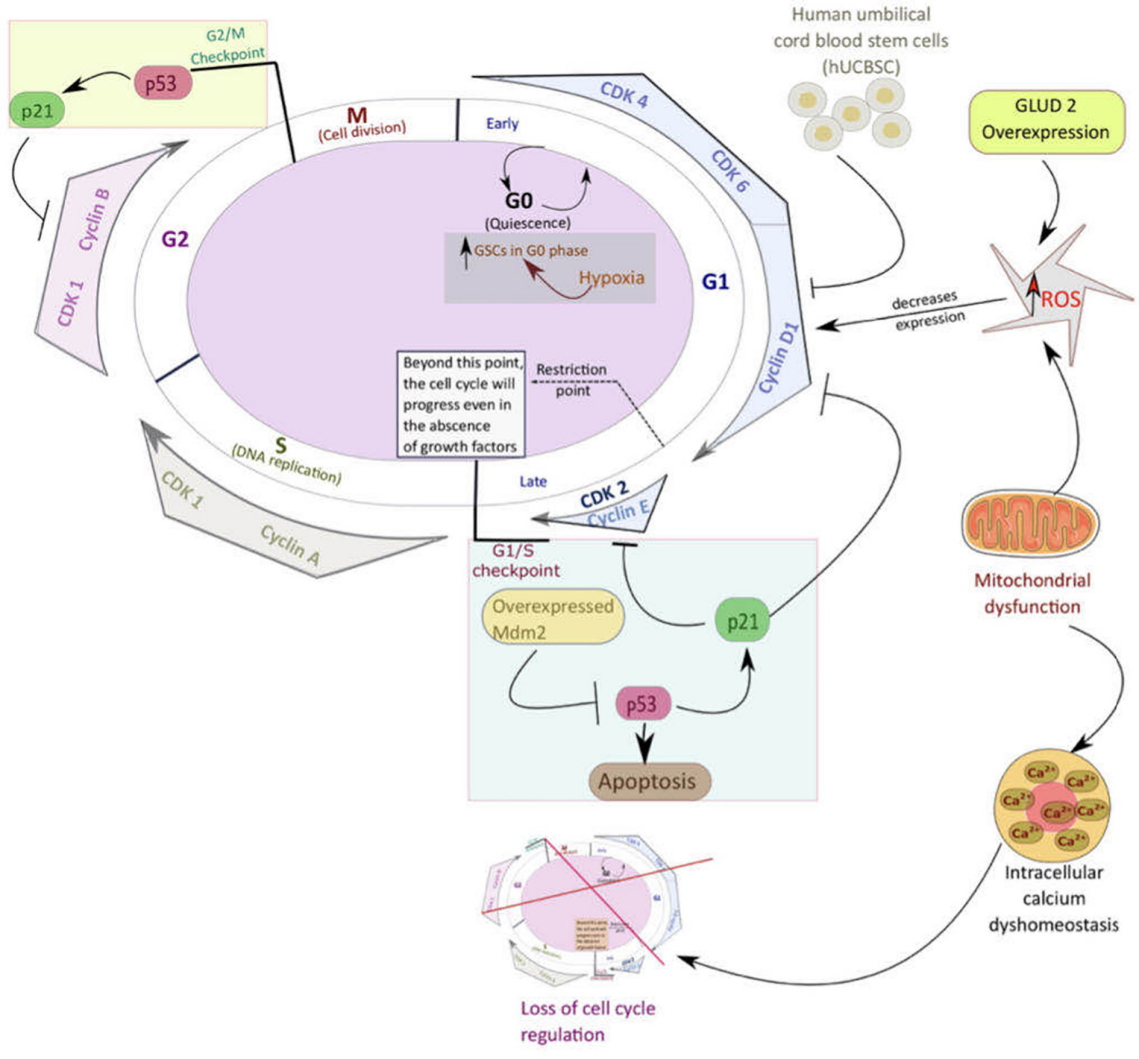
An overview of the pathways that mediate quiescence in glioblastoma stem cells (GSCs). Mitochondria play a significant role in critical cellular processes such as cell cycle control, cell metabolism, regulation of calcium homeostasis, and reactive oxygen species (ROS) generation. Like most other types of cancer stem cells, GSCs utilize mitochondria oxidative phosphorylation (OXPHOS) to keep up their increased proliferation, resistance, and stemness. Mitochondrial dysfunction enhances tumorigenesis through different pathways such as loss of cell cycle control, intracellular calcium dyshomeostasis, increased transition of GSCs into the quiescent state, and decreased apoptosis.

GSCs’ surface molecular biomarkers include CD49f^+^, CD90^+^, CD44^+^, CD36^+^, EGFR^+^, A2B5^+^, L1CAM^+^, and CD133^+^ (21). Glycerol-3-phosphate dehydrogenase 1 (GPD1) is another important marker that distinguishes GSCs from normal neural stem cells and can be used as a prognostic factor. Following chemotherapy, dormant GSCs, expressing GPD1 and mainly located at the GBM tumor borders, can be activated (22). Neural stem cells (NSCs) or transformed astrocytes might give rise to GSCs following gaining access to stem-specific transcriptional programs. GSCs are maintained through epigenetic regulators and modify the gene expression in response to external cues (7). Radiation enhances tumor recurrence due to tumor cell DNA mutations conferred by radiation, thus, rendering the tumor cells resistant to treatment. However, GSCs are not only able to survive the extensive course of chemoradiotherapy but can also promote radiotherapy resistance through the preferential activation of DNA damage checkpoint response that, in turn, promotes their DNA repair capacity. As shown in **Figure 2**, cell cycle checkpoints are critical regulators of cell proliferation and development. Quiescent GSCs express a higher amount of G0/G1-phase regulatory molecules such as cyclin D1, cyclin D2, and cyclin E) at the transcriptional and translational levels (23). Certain genes, such as ectonucleotidase ENPP1 (ectonucleotide pyrophosphatase/phosphodiesterase 1), are overexpressed in GSCs. Their function is usually related to stem cell feature maintenance, cell cycle control, cell death, and potential to proliferate (24).

Transcriptomic analyses of samples of recurrent and newly diagnosed GBM have shown that GSCs, locating in different regions of the tumor, are characterized by different degrees of stemness and gene expression pattern; however, this intratumoral heterogeneity is not random and depends on the intratumoral architecture. Studies have shown that harvesting four samples from a single tumor is sufficient to predict and optimize therapy outcomes. It is important to note that post-operative radiochemotherapy can further induce longitudinal changes in gene expression of GSCs. On the other hand, the limitation of performing biopsy after each round of therapy is another challenge for studying these longitudinal mutational alterations. These result in an increased resistance rate after each therapy session (25–27).

#### Metabolic Features of GSCs

Metabolic alterations are evident in GSCs. Though rapidly proliferating cells from GBM patients are glycolytic, only a small fraction of these are GSCs which are quiescent and capable of self-renewal (28). Self-renewing GSCs, similar to most other types of cancer stem cells, utilize both glycolytic and OXPHOS. To keep up their increased proliferation, rapidly proliferating GBM cells utilize glycolysis while quiescent GSCs depend on OXPHOS to maintain their stemness. Unlike previous speculation, GSCs can switch between different energy pathways and exhibit intermediate metabolic features to adapt their metabolism according to the different conditions such as environmental stressors such as radiation. Moreover, quiescent GSCs exhibit lower glycolysis and oxygen consumption and a much acidic extracellular space compared to the differentiated GBM cells (18, 19).

Specific features of GSCs such as GSCs’ self-renewal and decreased apoptosis are the hallmark of GBM resistance. Several factors regulate GSC proliferation and survival. One of the significant factors contributing to the increased tumorigenicity of GSCs is their high capacity for self-renewal. Early studies of mechanisms responsible for sustaining GSCs’ self-renewal property highlighted the importance of SRY-box transcription factor 2 (SOX2) gene expression. Commonly, SOX2 expression is upregulated during neural development and is essential in inducing pluripotency (29). However, its overexpression in GSCs is associated with increased tumorigenicity and resistance. Further experiments on tumor‐initiating cells (TICs) showed that SOX2 knockdown leads to decreased proliferation and self-renewal capacity. Moreover, these studies showed that GSCs share a similar mechanism with normal neural stem cells to sustain their stemness (30).

### GBM Tumor Constitution and the Surrounding Tumor Microenvironment

The hierarchical model proposed for GBM involves the progression from stem cell populations to more differentiated progeny (31). Single-cell RNA-seq (scRNAseq) studies of IDH mutant gliomas have shown glioblastoma trilineage hierarchy, including progenitor, neuronal, and astro-mesenchymal cancer cells, among which the progenitor cancer cells have the highest proliferative and lowest differentiated properties (32). Furthermore, in IDH wild type GBM cells, proliferating GSCs, referred to as “progenitor GSCs” display a more rapid growth rate and a higher chemoresistance property. Previously, several pathways, such as EZH2, FOXM1, and Wnt, associated with GSC self-renewal and tumorigenicity, have been identified. Recently, another critical pathway, the E2F4 pathway, has been identified by Couturier and colleagues. E2F gene family plays a key role as a cell cycle regulator and is critical for GSC progenitor cells. The inhibition of E2F4 is negatively correlated with GSC progenitor proliferation (33). In addition to the previously mentioned pathways, mitochondrial dynamics is crucial in regulating postmitotic cell fate. Iwata and colleagues showed that shortly after mitosis of neural stem cells, daughter cells that undergo and displays mitochondrial fusion maintain their self-renewal property, and those with mitochondrial fission differentiate into neurons (34). However, further studies are required to determine whether a similar mechanism exists in different GSC lineages.

As in normal tissues, quiescent and active CSCs coexist in the tumor bulk (35).GSCs consist of a small subpopulation of stem-like cells conferring tumor recurrence (36). Normal neural stem cells (NSCs) of the brain are located in the subventricular zone and hippocampus (37). Since GSCs’ surrounding microenvironment has to fit their need to maintain their stemness, intratumoral GSCs reside in specific locations such as perivascular, hypoxic, and necrotic niches. The perivascular niches can provide essential signals (such as Wnts) necessary for GSC maintenance, growth, and invasion (38–40).

Along with GSLCs and differentiated glioblastoma cells, other types of cells such as neural precursor cells (NPCS), astrocytes, neurons, macrophages, microglia, and endothelial cells as well as vascular components and extracellular matrix (ECM) are contributing to the intratumoral heterogeneity (41). Cellular components of the tumor communicate with each other and distant cells through extracellular vesicles (EVs). These EVs can also alter tumor growth, resistance, and death (42).

### Role of Mitochondria in GBM Tumorigenesis and Metastasis

Mitochondria, known to be responsible for cellular respiration, generation of oxidative radicals and their central role in apoptosis, DNA repair, autophagy, and cell cycle control, have recently been the focus of attention for the role of their genome in cancer development. The proposed role of mitochondria in tumorigenesis and metastasis has been studied in several types of malignancies, such as breast, lung, and prostate cancer. Mitochondrial dysfunction is associated with altered metabolism and can lead to enhanced tumorigenesis and metastasis. A broad study on mitochondrial cancer genome has shown that hypermutation, variations in structure and copy- number, and somatic transfer of mtDNA into the nuclear genome are associated with increased risk of cancer development and growth, and metastasis (43). Studies have shown that autophagy plays a critical role in the process of tumor cell survival, growth, and resistance. Different cancer therapeutic agents exert different regulatory effects on autophagy, leading to activation or inhibition of cytoprotective or cytotoxic autophagy. Moreover, in some types of malignancies such as GBM, chemoresistance to the first-line therapy agents such as TMZ can be mediated *via* ROS induced- activation of cytoprotective autophagy. Therefore, understanding the interplay between mitochondria, autophagy, tumor growth, resistance, and metastasis will provide us with better clues to new treatment strategies (44).

Mitochondria are responsible for maintaining the oxidant-antioxidant system in a cell. Oxidative damage, which has been implicated in tumorigenesis, usually follows mitochondria dysfunction. Mutations in genes encoding components of mitochondrial protein complexes such as NADH-ubiquinone oxidoreductase chain 4 (ND4) subunit can lead to elevated superoxide radical (O_2_^•–^) production, thus resulting in sustained ROS-dependent oncogenic pathways and induction of mitochondrial DNA (mtDNA). These changes are associated with an increased risk of tumorigenesis and metastasis in GBM (45).

GLUD2, which encodes for glutamate dehydrogenase (GDH), plays a critical role in regulating GBM tumorigenesis and is involved in normal cellular processes such as Krebs cycle and energy production as well as ammonia homeostasis (46). GDH is a mitochondrial enzyme, and its primary function is the reversible catabolization of glutamate to α-KG and ammonia. Typically, GDH exhibits high activity levels in specific mammalian organs such as the brain, liver, pancreas and kidney (47). Overexpression of GLUD2 is associated with the modification of mitochondrial function and metabolic profile of human GBM cells. GLUD2 overexpression is associated with increased ROS production due to increased mitochondrial oxidative metabolism and increased oxygen consumption levels (48). An increase in ROS levels causes cell cycle arrest in G0/G1 due to the decreased cyclin D1 and E expression (49). Also depicted in **Figure 2**, increased ROS levels inhibit the cell cycle’s progression, hence, causing cells to remain in their quiescent stage.

The Warburg effect, which is characterized by increased tumor cell glycolysis and decreased mitochondrial energy metabolism even in the presence of oxygen, can be seen in various malignancies such as GBM (50). Furthermore, malignant cells raise the mitochondrial apoptotic threshold by activating mitochondrial maintenance programs, which is important for enhancing cancer cell survival, proliferation, and metastasis. Other organelles such as the nucleus and endoplasmic reticulum and their crosstalk with mitochondria are essential components of cancer cell physiology such as survival, proliferation, metastasis, and stemness (51). In extreme environmental conditions such as hypoxia and acidic shift of the environment, nutritional deficiency and radiation, GSCs use specific protective mechanisms such as activation of stress response pathways to counteract the anti-cancer effects of endogenous stressors such as increased ROS production and exogenous stressors such as chemotherapy agents. These pathways, such as cytosolic heat shock response (HSR), the integrated stress response (ISR), and unfolded protein response (UPR), are either mediated by mitochondria or endoplasmic reticulum (ER) or cooperation of both organelles (52, 53).

### Glioblastoma Stem Cell Maintenance, Differentiation, and Quiescence

#### Stem Cell Maintenance

Stem cell maintenance is critical for GBM tumor recurrence, tumorigenicity, and metastasis. This stem cell feature is mediated through different mechanisms. It is noteworthy that differentiated GBM cells demonstrate lower therapy resistance compared to GSCs. The more we learn about these novel pathways, the better we can develop anti-cancer agents effectively targeting GSCs and induce their differentiation into the less resistant GBM cell types. GSCs employ specific mechanisms to maintain their stem cell features. One of these mechanisms is to counteract factors that can induce cell differentiation, such as bone morphogenetic proteins (BMPs). In response to anti-GSCs effects of BMP, GSCs secrete gremlin1, a BMP antagonist that inhibits BMP signaling, resulting in maintenance of stem cell features such as self-renewal capacity (54).

Hypoxia is another crucial factor that maintains and regulates stemness features and undifferentiated state in neural, hematopoietic stem cells, and GSCs (55, 56). Under hypoxic conditions, the number of GSCs in the G0 phase increases and more differentiated glioblastoma cells are induced into the undifferentiated form. Hypoxia maintains GSCs through the activation of NOTCH pathway, which is mediated by hypoxia-inducible factor-1α (HIF-1α) and 2α (HIF-2α) (56–58). Moreover, hypoxia can induce mixed-lineage leukemia 1 (MLL1), a histone methyltransferase, to increase the sensitivity and response of GSCs to hypoxia-induced regulation of stemness features (55).

An important tumor suppressor, p53 regulates different cellular functions such as cell differentiation, DNA repair, and angiogenesis. Mouse double minute 2 homolog (MDM2) gene is a negative regulator of p53. Within cells, p53 is usually present in low levels albeit, in certain types of malignancies, due to disrupted MDM2 and p53 interaction, p53 is upregulated to prevent cells’ malignant transformation in response to oncogenic stress (59). Conversely, in some malignancies such as GBM, MDM2 is overexpressed, and as a result, the activity of p53 is inhibited (60). In addition, Oliner et al. demonstrated the importance of MDM2 in maintaining GSC stemness, inhibition of which can cause further inhibition of factors related to GSCs stemness (61). Intriguingly, cholesterol might be involved in GSC stemness. RNA sequencing comparison of patient-derived GSCs and differentiated GBM cells showed the importance of cholesterol biosynthesis pathway in maintaining GSC stemness. More studies revealed that farnesyl diphosphate synthase (FDPS), which serves as an important enzyme in isoprenoid biosynthesis, has a vital role in GSC stemness maintenance (62). It is of note that GSCs highly express ectonucleotidase ENPP1 (ectonucleotide pyrophosphatase/phosphodiesterase 1) compared to other types of cells such as NSCs. Ectonucleotidase ENPP1 is involved in maintaining GSCs, and its knockdown induces GSCs to differentiate into GBM cells, lowers cellular proliferation rate, induces cell death, and decreases chemotherapy resistance (24).

Long non-coding RNAs (long ncRNA, lncRNA) are other essential mediators of GBM resistance, involved in various diseases and act as critical biological regulators. Follow-up of patients with GBM showed that overexpression of TP73-AS1, a GBM-associated lncRNA, maintains stemness of GSCs through interactions involving multiple pathways, thus leading to increased resistance of GBM cells to TMZ therapy (4). That said, lncRNA are good targets for potential therapeutic options.

#### Transformation of GSCs Into Differentiated Cells and Dedifferentiation of GBM Cells Into Stem-Like Cells

Early studies have established the importance of c-Jun N-terminal kinase (JNK) signaling pathway in GSC maintenance, self-renewal, and differentiation. Activation of the JNK pathway is necessary for self-renewal and inhibition of GSC from differentiation. Therefore, JNK pathway inhibition promotes GSC differentiation and diminishes tumor-initiating potential, making them more prone to cancer therapy strategies (63). Nutritional stress, acidic environment, and hypoxia induce dedifferentiation of GBM cells into GSCs. However, eliminating any of these conditions permit GSCs proliferation and transition into differentiated GBM cells, with increased sensitivity to the anti-cancer therapy (23).

Dedifferentiation of GBM cells into stem-like cells, possible through various mechanisms, is required for tumor continuity and is usually associated with a low survival rate. As we previously mentioned, hypoxia can induce transformation of differentiated GBM tumor cells into an undifferentiated state that exhibits stem-cell-like features. The tumor microenvironment plays a critical role in the stemness and differentiation state of different tumor cells. Cancer therapy, such as irradiation, can alter the tumor microenvironment and promote stem-like cell features, angiogenesis, recruitment of inflammatory cells such as Ly6G^+^ inflammatory cells like tumor-associated neutrophils (TANs) and granulocytic myeloid-derived suppressor cells (G-MDSCs) (64). Following radiation therapy, GBM tumor cells are driven to dedifferentiation. Besides, Ly6G^+^ inflammatory cells further promote the secretory feature of senescent GBM cells and alteration of tumor microenvironment, which are mediated through NFκB signaling pathway. Ly6G^+^ inflammatory cells promote GBM tumor cells dedifferentiation through the NO-ID4 axis. Inhibitors of differentiation (ID) family members are important regulators of GSCs with stem-like features and GBM cells’ transformation into GSCs (65–68).

Nutritional stress or nutritional deprivation instigates dedifferentiation of GBM cell into GSCs and is associated with an increased expression of GBM stem-like cell features, including biomarkers such as CD133, therapy resistance, and angiogenesis. Moreover, nutritional stress activates Wnt and Hedgehog signaling pathways and causes overexpression and nuclear localization of stemness markers such as Sox2, Oct 4, and Nanog at the transcriptional and translational levels (23).

#### Transition of GSCs Into Quiescence and Mechanisms Involved in Quiescent State Maintenance

The transition of GSCs into quiescent state (G0–G1 phase arrest) is a tumor protective response following chemoradiotherapy. Proteins such as Cdk 4, Cdk 6, cyclin B1, and cyclin D1 regulating the cell cycle are down-regulated upon entry into the quiescent state. Inhibition of Cyclin D1, which regulates cell cycle progression through the G1 phase in human umbilical cord blood stem cells (hUCBSC), can induce glioblastoma cell lines to enter cell cycle arrest (69). A decrease in intracellular pH is associated with GSCs induction into the quiescent state, increased stemness and increased expression of stemness markers (20). Though it was proposed that the simultaneous treatment with TMZ and glucose starvation could promote GBM tumor cell death, a recent study by Wang et al. suggested that glucose starvation can induce resistant GBM tumor cells to enter quiescence, thus leading to their increased resistance to chemotherapy (70).

Quiescent GSCs stay in a functional reversible G0 phase, vigorously maintained by several pathways until reactivation and reentry into the cell cycle. In **Figure 2**, various pathways involved in GBM quiescence are illustrated. BMP signaling, found to be the mediator of GSCs quiescence, is further regulated by its downstream targets, ID1 and p21, and is also associated with increased chemoradiotherapy resistance. A series of experiments by Sachdeva and colleagues showed that BMP4 not only modulates GSC phenotype but also causes an inhibition of GSC self-renewal capacity and tumorigenicity (71). In recent years, mitochondria have been recognized as a crucial regulator of GSC quiescent state maintenance, potentially serving as an important target against GBM resistance.

As aforementioned, mitochondria can counteract the destructive effects of endogenous and exogenous stressors in GSCs. One of these mechanisms is the activation of mitochondrial stress pathways such as mitochondrial unfolded protein response (UPR^mt^). Chaperones and proteases of the UPR^mt^ pathway maintain cellular homeostasis through proteotoxic stress elimination. Intracellular calcium ion (Ca^2+^) homeostasis, regulated by mitochondria, is necessary as intracellular Ca^2+^ modulates cell-cycle progression (72). Mitochondrial Ca^2+^ uptake and regulation of store-operated Ca^2+^ entry (SOCE) activity controls Ca^2+^ levels through store-operated channels (73, 74).

#### Reactivation of Quiescent GSCs

Quiescent GSCs reside mainly in pre-necrotic areas of the tumor. Upon removal of exogenous and endogenous stressors, GSCs reactivate and migrate into the oxygen and nutrient-rich areas such as perivascular zones for proliferation and differentiation. Nevertheless, how these cells get reactivated and enter the proliferative phase is yet to be clarified.

GINS complex, a heterotetrameric complex which consists of four subunits including Sld5, Psf1, Psf2, and Psf3, is important in initiating DNA replication and progression by serving as a DNA helicase in association with CDC45 and MCM2-7 (75, 76). Recent studies have shown that induction of GINS expression is not only required for the reactivation of quiescent GBM cells residing in peri-necrotic niches, but also determines the proliferative phenotypes of quiescent GBM cells. Quiescent GSCs show decreased GINS protein subunit levels, which positively correlate with the results stating that GINS is involved in the reactivation of quiescent GSCs (77).

### Therapeutic Implications of Proliferative and Quiescent GSCs

A significant hurdle in GBM treatment is the presence of resistant intratumoral GSCs. Most current treatment strategies show little to no efficacy due to the evasiveness of GSCs. However, engineered oncolytic viruses are a promising treatment strategy for some malignancies, such as GBM. Recent discoveries have shown that the Zika virus (ZIKV; the primary cause of newborn microcephaly outbreak in 2015) could treat resistant GBM. The Zika virus primarily kills different brain cells, such as neural precursor cells (NPC), leading to microcephaly. Further studies have shown that ZIKV displays higher oncolytic activity toward GSCs than NPCs and differentiated glioblastoma cells, and at the same time, causes no harm to normal brain tissue. ZIKV confers its oncolytic property by inhibiting the self-renewal capacity of GSCs (78). Earlier studies demonstrated that the upregulated expression of SOX2 in GSCs is associated with GBM’s higher tumorigenicity due to an increased self-renewal capacity (30). SOX2 acts by modulating GSCs ZIKV infection and regulating their expression of the Integrin α_v_ subunit. Integrin α_v_ plays a major role in cellular migration, proliferation, and intracellular signaling by the formation of a heterodimer with one of the distinct β subunits including β_1_, β_3_, β_5_, β_6_, and β_8_ (79). Further experiments showed that α_v_β_5_ plays a critical role in ZIKV infection of GSCs by maintaining the GSCs (80).

Manipulation of GSC differentiation and proliferation can serve as an important target for effective treatment of resistant GBM. Theoretically, each GSC has three choices: self-renewal to produce two GSCs, asymmetric division to produce one GSC and one cell that proliferates but cannot self-renew, and commitment to differentiate to produce two cells that proliferate but cannot self-renew. Generally, GSC are present in two different niches: quiescent and active in cell division. Differentiated GBM cells exhibit a lower resistance to chemoradiotherapy compared to undifferentiated and quiescent cancer stem cells. GSCs exhibit low expression levels of MKP1, which is a dual-specificity phosphatase and negatively regulates ERK1/2 and p38 MAPK. The role of MKP1 is significant since the high expression level of MKP1 is associated with the differentiation of GSCs and their increased sensitivity to TMZ (81). What makes these findings significant is that a group of glioblastoma patients with a higher expression level of MKP1 showed improved prognosis and overall survival rate. Studies on histone deacetylase inhibitors (HDACIs) showed that these agents could cause an upregulation of glioma cell MKP1; thus, MKP1 is a promising treatment strategy targeting resistant GSCs (82).

Another critical target of resistant GBM therapy is mitochondria. As aforementioned, mitochondria play a critical role in tumor biology by regulating cell cycle, metabolism, apoptosis, DNA repair, and maintenance of stemness in cancer stem cells. Mitochondria enable cancer cells to be more tolerant against hypoxia, radiation, and cytotoxic agents by activating stress response pathways and altering cell metabolism. A small synthetic molecule named KHS101 was discovered to effectively impair mitochondrial heat shock protein family D member 1 (HSPD1) and its dependent metabolic pathway. KHS101 is a good anti-tumor agent since it can effectively exert its anti-tumor effect on different subtypes of cancer cells, including GSCs, without negatively impacting intact cells. KHS101 interrupts GBM cell aerobic glycolysis and mitochondria respiration-dependent pathways and causes aggregation of HSPD1 and metabolic enzymes in GBM cells, thereby promoting their metabolic exhaustion. Induction of acute metabolic stress, which leads to alteration of the cell cycle, metabolic, and stemness pathways, causes different subtypes of GBM cells to undergo autophagy and apoptosis after KSH101 treatment. All of this could lead to the loss of stem cell-like features of GBM cells and an increase in cell death. Further experiments on patient-derived tumor xenografts in mice showed that KHS101 treatment could successfully diminish tumor growth and increase the survival rate (83).

Induction of reactive oxygen species (ROS), by-products of mitochondrial metabolism, can be used as another effective treatment strategy. In GSCs, ROS is present at low levels due to the free radical scavenging system. Moreover, low levels of ROS are associated with a higher malignant potential. Further, studies have shown that high ROS levels can prevent the cancer progression (84, 85). Curcumin, the main component of turmeric, has previously shown its antioxidant effects on the prevention and progression of different types of cancers. However, what makes curcumin an even more valuable anti-cancer agent is that it can target non-GSCs (GBM cell that do not have GSCs) and effectively target GSCs through different mechanisms such as the induction of mitochondrial ROS, leading to MAPK activation, STAT3 inactivation, and downregulation of STAT3 targets. Together, these mechanisms could decrease the self-renewal and survival of GSCs and non-GSCs (86).

Previously, we stated that p53 inactivation due to MDM2 overexpression could lead to GBM tumor recurrence *via* the absence of inhibition of stemness-related factors in GSCs. Experiments on patient-derived GSCs have shown that GBM stemness can be inhibited by MDM2 inhibitor, AMG232. However, p53 reactivation is required to increase the sensitivity of GBM tumor cells to MDM2 inhibitors (61). Besides, targeting the cholesterol biosynthesis pathway has shown to be a promising treatment strategy against resistant GSCs. Alendronate, a popular anti-osteoporotic agent, is effective in GBM treatment as it inhibits farnesyl diphosphate synthase (an enzyme involved in isoprenoid biosynthesis and GSCs’ maintenance) that in turn, reduces embryonic stem-cell features and activation of pathways related to necrosis and development in GBM cells (62).

Lack of selectivity of specific agents to target GSCs is another obstacle in managing GBM. Recently, an RNA aptamer (a shortened form of aptamer 40L) known as A40s, was developed to bind to CD133^+^-GSCs selectively. Moreover, GSCs can internalize these aptamers, which could be used as a means of drug delivery such as microRNAs targeting and inhibiting GSCs (87). Induction of apoptosis through mitochondria ROS formation is an important mechanism employed by certain agents such as sulforaphane, which is an isothiocyanate found in cruciferous vegetables that exhibits anti-cancer properties (88).

The few aforementioned therapeutic strategies could potentially be used in the management of GBM. However, none of these therapeutic agents can achieve effective GBM treatment. An effective therapeutic strategy would be one that prevents GBM progression, recurrence, and reduces the possibility of GBM resistance development. An ideal therapeutic agent should possess specific characteristics such as high affinity to its target cells (GSCs), reasonable price, public availability and, most importantly, effective against GSLCs and quiescent GSCs.

## Discussion

GSCs are a distinct subpopulation of GBM cells with unique self-renewal properties, the potential to proliferate and differentiate. In the presence of environmental stressors (such as chemoradiotherapy, nutritional deprivation, hypoxia, and acidic shift of the environment), these cells undergo cell cycle arrest and become quiescent. The quiescent state is phase G0 of the cell cycle, where cell inactivity is observed. Chemoradiotherapy mostly affects rapidly dividing cells, which explains why the quiescent state protects GSCs during chemoradiotherapy. GSCs are dormant until an activating signal causes them to reactivate, and migrate to the perivascular regions, which provides them with enough nutrition and oxygen for proliferation. Currently, we are faced with several obstacles in the effective treatment of GBM such as the presence of quiescent GSCs. Conventional therapies lack specificity for quiescent GSCs, making it difficult to eradicate these tumor-driving cells. Although extensive research has identified most of the pathways and mechanisms involved in quiescent state activation and reactivation in other kinds of malignancies and neural stem cells, understanding of quiescent GSCs is not well elucidated, therefore, more studies are warranted.

For effective GBM treatment, future therapeutic strategies focusing on reducing GSC transition into the quiescent state and reactivation of existing quiescent GSCs, might be beneficial. Another focus could be induction of GSCs to proliferate and become committed to differentiate. This way, the tumor-initiating cell population could be significantly reduced. More research on the specific role of the brain lymphatic and immune system in GBM and the interaction between these systems is also warranted. It is necessary to understand how proliferative GBM cells and quiescent GSCs behave in different microenvironments, including in an inflammatory setting.

Methods such as High-throughput Automated Single Cell Imaging Analysis (HASCIA) facilitate the assessment of heterogeneity and state transition in GSCs at the single-cell level, which is vital for future GBM research and discovery of new anti-cancer drugs that can target state transitions, for instance, inhibition of quiescent state transformation or activation of differentiated state (89). A thorough understanding of GSC transition between quiescent, self-renewing and proliferative progenitor states that cannot self-renew could help develop targeted therapy to these specific populations with little influence on the normal neural stem cells. We believe that this ideal therapy will most likely be a combinational therapy due to the complexity of the GBM hierarchy.

## Author Contributions

YI, BJ, and FO contributed equally and thus share the first authorship. CS, JL, JW, and ZD made sure this article was up to standard. CS is the corresponding author and JL is the co-corresponding author. All authors contributed to the article and approved the submitted version.

## Funding

This project was supported by Key Project of Ministry of Science and Technology of China: Project No.: 2018YFA0108603 and Zhejiang Natural Science Foundation Project: Project No.: LY17H160016.

## Conflict of Interest

The authors declare that the research was conducted in the absence of any commercial or financial relationships that could be construed as a potential conflict of interest.
